# The correlation between three teleconnections and leptospirosis incidence in the Kandy District, Sri Lanka, 2004–2019

**DOI:** 10.1186/s41182-021-00325-z

**Published:** 2021-05-26

**Authors:** N. D. B. Ehelepola, Kusalika Ariyaratne, A. M. S. M. C. M. Aththanayake, Kamalanath Samarakoon, H. M. Arjuna Thilakarathna

**Affiliations:** 1 The Teaching (General) Hospital–Peradeniya, Peradeniya, Sri Lanka; 2 Lanka Hydraulic Institute, Moratuwa, Sri Lanka; 3 Faculty of Engineering, University of Peradeniya, Peradeniya, Sri Lanka

**Keywords:** Leptospirosis, Teleconnections, ENSO, IOD, ENSO Modoki, Sri Lanka, SSTA, Wavelet analysis, DCCA

## Abstract

**Background:**

Leptospirosis is a bacterial zoonosis. Leptospirosis incidence (LI) in Sri Lanka is high. Infected animals excrete leptospires into the environment via their urine. Survival of leptospires in the environment until they enter into a person and several other factors that influence leptospirosis transmission are dependent upon local weather. Past studies show that rainfall and other weather parameters are correlated with the LI in the Kandy district, Sri Lanka. El Ni*ñ*o Southern Oscillation (ENSO), ENSO Modoki, and the Indian Ocean Dipole (IOD) are teleconnections known to be modulating rainfall in Sri Lanka. There is a severe dearth of published studies on the correlations between indices of these teleconnections and LI.

**Methods:**

We acquired the counts of leptospirosis cases notified and midyear estimated population data of the Kandy district from 2004 to 2019, respectively, from weekly epidemiology reports of the Ministry of Health and Department of Census and Statistics of Sri Lanka. We estimated weekly and monthly LI of Kandy. We obtained weekly and monthly teleconnection indices data for the same period from the National Oceanic and Atmospheric Administration (NOAA) of the USA and Japan Agency for Marine-Earth Science and Technology (JAMSTEC). We performed wavelet time series analysis to determine correlations with lag periods between teleconnection indices and LI time series. Then, we did time-lagged detrended cross-correlation analysis (DCCA) to verify wavelet analysis results and to find the magnitudes of the correlations detected.

**Results:**

Wavelet analysis displayed indices of ENSO, IOD, and ENSO Modoki were correlated with the LI of Kandy with 1.9–11.5-month lags. Indices of ENSO showed two correlation patterns with Kandy LI. Time-lagged DCCA results show all indices of the three teleconnections studied were significantly correlated with the LI of Kandy with 2–5-month lag periods.

**Conclusions:**

Results of the two analysis methods generally agree indicating that ENSO and IOD modulate LI in Kandy by modulating local rainfall and probably other weather parameters. We recommend further studies about the ENSO Modoki and LI correlation in Sri Lanka. Monitoring for extreme teleconnection events and enhancing preventive measures during lag periods can blunt LI peaks that may follow.

**Supplementary Information:**

The online version contains supplementary material available at 10.1186/s41182-021-00325-z.

## Background

Leptospirosis is an emerging zoonotic disease caused by pathogenic and opportunistic spirochete bacteria belonging to the genus *Leptospira* [1–5]. A small fraction of infections result in severe leptospirosis (Weil’s disease) [5]. Severe leptospirosis results in more deaths globally mostly in the tropics than other hemorrhagic fevers and a large percentage of those are in the prime age of their working life [6]. Leptospirosis may be the most widely distributed zoonosis in the world and leptospirosis incidence (LI) in Sri Lanka is among the highest in the world; hence, it is a major public health problem of people and domestic animals in Sri Lanka [7, 8]. Leptospirosis was first confirmed in a Sri Lankan patient in 1959, became a notifiable disease in 1991, and now highly endemic in many districts including Kandy District. In Kandy, LI shows an annual rise during October–December and in some years spikes during April–July. Outbreaks occur once in several years, such as in 2008 [5].

### The life cycle of the pathogen and how weather affects it

Infected animals excrete leptospires into the environment via their urine. Most of the time, people get infected by leptospires in damp soil or surface water bodies through breaches of the skin or through mucus membranes [4, 5]. Survival of leptospires in the environment until they enter into a host, biofilm making by leptospires that influences the load of infecting bacteria, depends upon several local weather parameters including local rainfall, temperature, humidity, evaporation rate, and duration of sunshine [3]. Overflows after heavy rains disperse leptospires [1, 3, 5]. Among non-weather factors that affect the survival of leptospires, pH, salinity, texture, and microbiota of the soil are also modulated by local weather [1, 3]. Outdoor manual work exposing to damp soil and water, especially working in rice paddies, is a common risk factor of exposure to leptospirosis in Sri Lanka and in other tropical countries [1–3, 5, 8]. Field preparation and harvesting of many crops especially rice are dependent on local weather. Rodents are a main reservoir of leptospires in nature and rodent population size, their activity, and percentage of leptospirosis-infected rodents fluctuate with local weather changes [1–5]. All in all, leptospirosis is a weather-sensitive infection.

### Description of teleconnections their indices

El Ni*ñ*o southern oscillation (ENSO), ENSO Modoki, and the Indian Ocean dipole (IOD) are the teleconnections relevant to the present study. ENSO is a coupled ocean-atmosphere phenomenon. Aperiodic oscillations of sea surface temperature (SST) in the Eastern and Western sides of the equatorial Pacific Ocean with associated atmospheric pressure oscillations are described as ENSO [9]. ENSO modulates weather in many parts of the world including Sri Lanka [10, 11]. ENSO has three phases. When SST of the Eastern equatorial Pacific Ocean is higher than usual, it is called the warm phase of ENSO (El Niño); the opposite is called the cold phase (La Ni*ñ*a) and there is an in-between neutral phase [9–11]. Nino indices indicate the phase and magnitude of ENSO [9]. Nino 1, 2, 3, 3.4, and 4 are different computations of SST anomalies (SSTA) between different selected locations of the Eastern and Western sides of the equatorial Pacific Ocean. Considering geographical proximity, Niño 3.4 and 4 are more relevant to the weather of Sri Lanka. The Southern Oscillation Index (SOI) is the index of coupled atmospheric pressure oscillations of ENSO [9]. The SOI indicates the surface atmospheric pressure difference between Tahiti (in the Pacific) and Darwin, Australia (on the Indian Ocean). The Multivariate ENSO Index (MEI) is a more lately developed blended index combining five variables of both SST and atmospheric components of the ENSO [9, 12]. ENSO usually accounts for the largest proportion of the inter-annual variation in climate in the world [9]. ENSO Modoki is a more recently discovered coupled ocean-atmosphere phenomenon. During its warm phase (El Ni*ñ*o Modoki), the central equatorial Pacific Ocean becomes warmer with colder Western and Eastern flanks with coupled atmospheric pressure changes. During the cold phase (La Ni*ñ*a Modoki) the central equatorial Pacific Ocean becomes colder with warmer Western and Eastern flanks [13]. The El Ni*ñ*o Modoki Index (EMI) calculated reflecting these SSTA is the index of the ENSO Modoki.

The Indian Ocean Dipole (IOD) is a coupled ocean-atmosphere phenomenon related to the tropical Indian Ocean. Higher SST in Western than the South Eastern tropical Indian Ocean and coupled atmospheric pressure changes is the positive phase of IOD and vice versa is the negative phase [14]. The Dipole Mode Index (DMI) computed reflecting this SSTA is the index of the IOD. IOD is also known to influence weather in Asia including Sri Lanka, Eastern Africa, and Australia [10]. Atmospheric changes of ENSO, ENSO Modoki, and IOD affect the weather of non-contiguous geographical regions. Therefore, they are called teleconnections. The net effect of these three teleconnections on local weather of Kandy/Sri Lanka is a result of their interactions with several other atmospheric phenomena like the two monsoons and subjected to modulation by the local geography especially by the central hill country of Sri Lanka where Kandy is situated [15].

### Reasons behind the present study

Teleconnections’s influence on climate-sensitive diseases has been gaining the interest of the medical community during the recent past [9]. However, our literature survey did not find any long-term studies on ENSO Modokis’ effects of LI in the English literature, and to the best of our knowledge, there is only one long-term published study regarding the correlation between an index of IOD and LI from anywhere in the English literature [3]. There is only one long-term study about ENSO’s influence on LI from South Asia (where Sri Lanka is) in the English literature [3].

Past studies have demonstrated ENSO, ENSO Modoki, and IOD’s modulation of rainfall in Kandy district and Sri Lanka [10, 15, 16]. We have illustrated how rainfall and other meteorological factors influence LI of the Kandy district [5]. All above considered, mechanistically, there are strong reasons to expect the LI of Kandy to be correlated with ENSO, ENSO Modoki, and IOD. Thus, we decided to study the correlation between indices of ENSO, ENSO Modoki, and IOD and leptospirosis incidence of the Kandy district for 2004–2019.

There is no accord in the scientific circles as to which ENSO index best describes ENSO phases [9]. We intended to compare the magnitudes of correlations between abovementioned teleconnection indices and LI to find which one of them has the strongest correlation with the LI of Kandy. As far as we know, there are no studies in the English medical literature that compare the magnitude of correlation of SSTA indices and coupled atmospheric change index of ENSO (SOI) between the incidence of any weather-sensitive disease to find the one with the best correlation with disease incidence.

We employed two analysis methods: wavelet time series analysis (wavelet analysis) and the time-lagged detrended cross-correlation analysis (time-lagged DCCA) method. Both methods are apposite to detect nonlinear and non-stationary correlations like those between teleconnection indices and weather-sensitive infectious disease incidences. Wavelet analysis is an established method used in similar past studies [3, 17–19]. We could not calculate the magnitudes of correlations with wavelet analysis. Hence, we used the time-lagged DCCA method to determine the magnitudes of correlation for comparing them and to cross-check wavelet results. We did not come across any past study using the time-lagged DCCA method to study teleconnections and LI correlation in the English literature.

## Methods

### Study setting

**Fig. 1:**
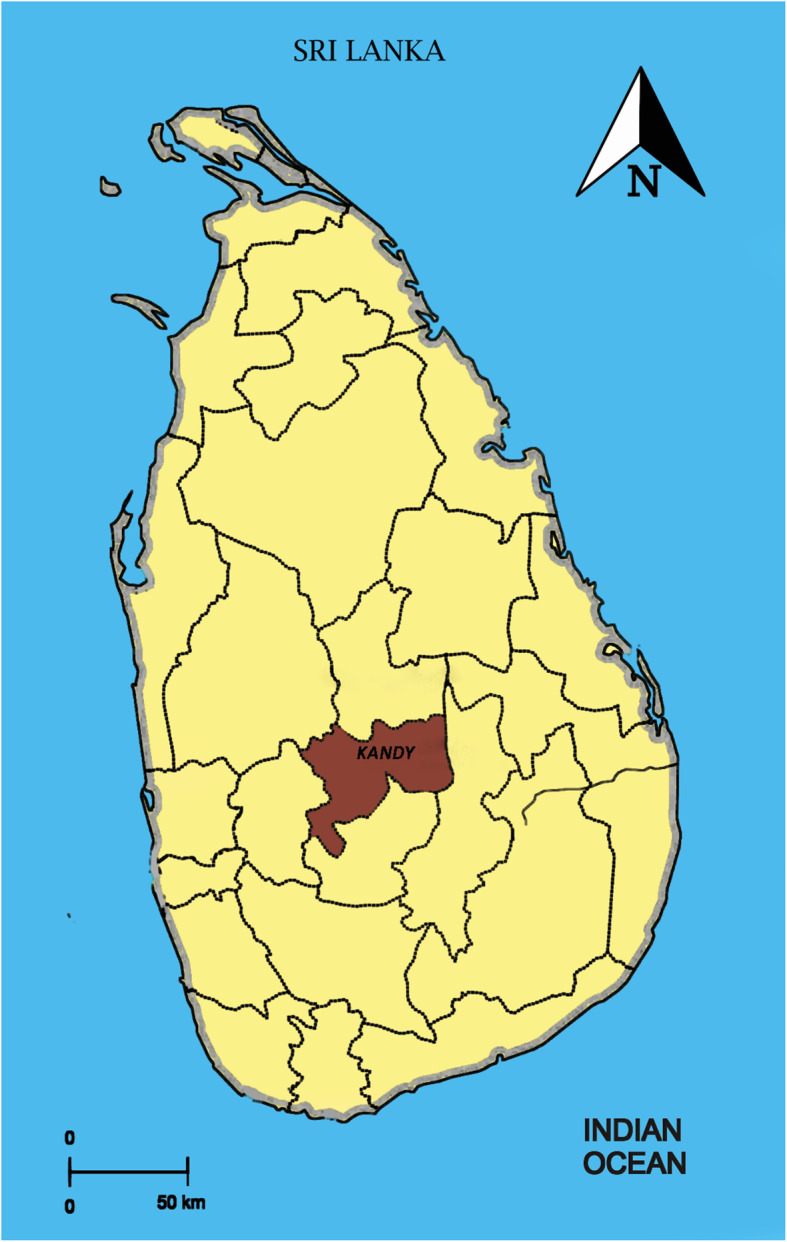
Our study area, Kandy district, on a map of Sri Lanka. Kandy district is shaded in light brown color in contrast to the other 24 districts of Sri Lanka

Kandy district is situated in the central hill country of Sri Lanka and has an area of 1,940.3 km^2^. The city of Kandy is in the center of the district. The estimated population of the district in 2015 was 1,416,000. Our study area is shown in a map of Sri Lanka in Fig. 1.

### Objectives and hypotheses

Our hypothesis was indices of ENSO, ENSO Modoki, and IOD are correlated with the LI of Kandy. Our objective was to determine the correlation patterns (with the lag periods) and their magnitudes between the LI of Kandy and indices of ENSO, ENSO Modoki, and IOD for the 2004–2019 period and to establish which teleconnection has the most influence on the LI of Kandy, also to determine which index of ENSO has the strongest correlation with the LI of Kandy. We envisioned exploring potential ways to use knowledge gained for improving leptospirosis control. We looked for a correlation between weekly/monthly LI and weekly/monthly Niño 3.4, Niño 4, EMI, and DMI SSTA indices. We looked for a correlation between monthly LI and monthly SOI and MEI indices as well.

### Data

We have used secondary data. We acquired the counts of leptospirosis cases notified from the Kandy district each week from the weekly epidemiology reports of the Ministry of Health of Sri Lanka from 2004 to 2019. Leptospirosis cases data of 2 weeks were missing. Missing data is filled with assuming a linear fit based on the surrounding available data before wavelet analysis. Since there are a total of about 834 weekly data points, and only two are missing, we believe filling the missing data with linear fit does not affect the final results. It is impossible to perform DCCA analysis with missing values. Hence, we replaced missing values with the averages of the previous four data points (MA(4)). The source of annual estimated mid-year population of the Kandy district for the same period was the Sri Lanka Department of Census and Statistics. Weekly Niño 3.4 and Niño 4 SSTA indices, plus monthly SOI, and MEI anomaly indices were obtained online from the National Oceanic and Atmospheric Administration of the United States (NOAA of the USA) [20]. Weekly DMI anomaly data and monthly EMI data were kindly provided by Dr.Takeshi Doi of the Japan Agency for Marine-Earth Science and Technology (JAMSTEC). Please see our data accessibility statement for further details. There were no missing data in Niño 3.4, Niño 4, and DMI, MEI, SOI, and EMI time series for our period of study.

## Analysis

We have estimated weekly leptospirosis incidence (LI) per 100,000 population for the 2004–2019 period. We converted weekly LI to monthly LI for time-lagged DCCA analysis. We determined correlation patterns with lag periods between weekly or monthly LI and Niño 3.4, Niño 4, and EMI SSTA indices and DMI SSTA index. We looked for correlation patterns including lag periods between monthly LI and monthly SOI and MEI.

### Wavelet analysis

We followed the same wavelet analysis methodology as in two past published studies by us, explained in detail in the methodology section of those open access papers available online, using MATLAB R2013a software of MATLAB Corporation, USA [3, 19]. Nevertheless, we like to highlight key points of the methodology here. We select an appropriate window and it is shifted along the signal in a time series, and for every position, the wavelet spectrum is calculated. The same process is then repeated many times with a slightly shorter and longer window for every new cycle. With wavelet transform, the product will be a collection of time-frequency representations of the signal with different resolutions. Cross-wavelet transform (XWT) and wavelet coherence (WTC) are used for examining relationships in time-frequency space between two time series. Continuous wavelet transform (CWT) is a common tool for analyzing localized intermittent oscillations in a time series. CWT is usually preferable to study two time series together that are expected to be related. The XWT detects regions in time-frequency space with high common power. WTC is the square of the cross-spectrum normalized by the individual power spectra. WTC gives a quantity between 0 and 1 and measures the cross-correlation between two time series as a function of frequency.

### Time-lagged detrended cross-correlation analysis

Autocorrelation is used to measure the correlation within the same signal and cross-correlation is used to determine the time-lagged correlation between two different signals. Similarly, detrended cross-correlation analysis (DCCA), proposed by Podobnik et al [21], is used to measure the correlation between two non-stationary series. This DCCA coefficient is defined for each scale of analysis *ν* as the ratio

$$ {\sigma}_{DCCA}(v)=\frac{F_{DCCA}^2(v)}{F_{DFA,x}(v){F}_{DFA,y}(v)} $$

where ${F}_{DCCA}^2(v)$ is a detrended covariance between partial sums of two non-stationary series {*X*_*t*_} and {*Y*_*t*_} for a window size *v* , *F*_*DFA*, *x*_(*v*) and *F*_*DFA*, *y*_(*v*) are detrended variances of partial sums of two series {*X*_*t*_} and {*Y*_*t*_}, respectively, for a window size *v*. The DCCA coefficient varies between −1 ≤ *σ*_*DCCA*_ ≤ 1. Similar to the standard correlation coefficient, *σ*_*DCCA*_ = 1 indicates that two non-stationary series {*X*_*t*_} and {*Y*_*t*_} are perfectly cross-correlated while *σ*_*DCCA*_= − 1 means two time series are perfectly inversely cross-correlated (anti-cross-correlated) [22–24].

Past lags of time series *x* may relate to series of *y*_*t*_. Based on DCCA, time lagged DCCA is developed to measure the strength of time-lagged cross-correlations between two non-stationary time series at different time lags [25]. It recognizes that the largest correlation was at which lags of the *x*-variable and it may support to predict *y*_*t*_. We used R statistical software to carry out time-lagged detrended cross-correlation analysis.

In R statistical software, DCCA is defined as the detrended cross-correlation between *x*_*t*_ and *y*_*t*_. Furthermore, the time-lagged detrended cross-correlation is calculated between *x*_*t*_ and *y*_*t* + *h*_ for *h* = 0, ± 1, ± 2, ± 3, . . ± *n* using R software. Negative *h* values give the correlation between *y*_*t*_ series at a time before *t* and *x*_*t*_ series at time *t*. For example, when *h* =  − 3, the value of time-lagged DCCA would give the correlation between *x*_*t*_ and *y*_*t* − 3_. Moreover, when we have predictors of *y*_*t*_ with negative *h*, it is said to be *x* lags *y* while positive *h* said that *x* leads *y*. In practice, it is important to recognize which series is leading and which series is lagging at a particular time lag. In this study, we will examine the *x*_*t*_ series (including its lags) to be a leading series of the *y*_*t*_series since we need to identify the patterns of *y* occurred due to *x* series. Hence, positive values of *h* on the time-lagged DCCA plot are examined. Time-lagged correlation between LI and six teleconnection indices were graphically identified and the strengths of the relationships are measured in this study. The function “rhodcca” under the “DCCA” package in R statistical software is used for the analysis, and further, package “ggplot2” is used for graphical illustrations.

## Results

The mean and median annual notified LI for Kandy district for our study period respectively were 9.1 and 7.0 per 100,000 population. LI was lowest in 2004 (3.0/100,000) and highest in 2008 (33.9/100,000).

**Fig. 2:**
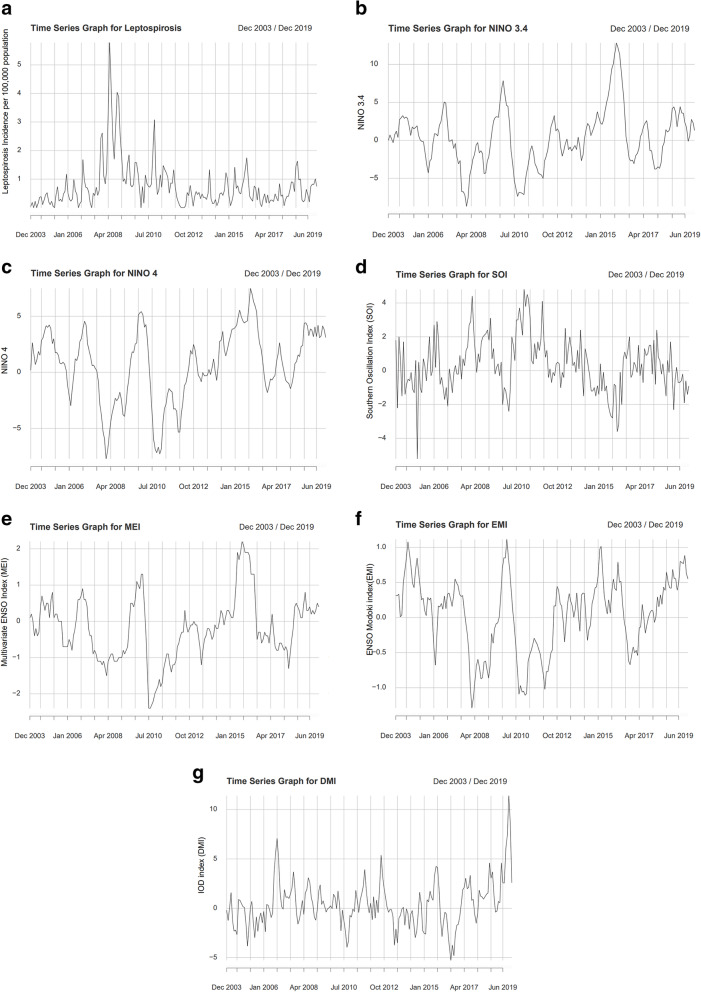
Time series graphs of the leptospirosis incidence and indices of the three teleconnections for 2004–2019. Panel **a**: Time series graphs of the monthly leptospirosis incidence (per 100,000 population) during the course of the year, for 2004–2019. *x*-axis: Time (months)/*y*-axis: monthly leptospirosis incidence (per 100,000 population). Panel **b**: Time series graphs of the monthly Niño 3.4 SSTA for 2004–2019. *x*-axis: Time (months)/*y*-axis: monthly Niño 3.4 SSTA. Panel **c**: Time series graphs of the monthly Niño 4 SSTA for 2004–2019. *x*-axis: Time (months)/ *y*-axis: monthly Niño 4 SSTA. Panel **d**: Time series graphs of the monthly SOI for 2004–2019. *x*-axis: Time (months)/*y*-axis: monthly SOI. Panel **e**: Time series graphs of the monthly MEI for 2004–2019. *x*-axis: Time (months)/*y*-axis: monthly MEI. Panel **f**: Time series graphs of the monthly EMI for 2004–2019. *x*-axis: Time (months)/*y*-axis: monthly EMI. Panel **g**: Time series graphs of the monthly DMI for 2004–2019. *x*-axis: Time (months)/*y*-axis: monthly DMI

Figure 2 is a collection of time series graphs of the LI and three teleconnection indices for 2004–2019.

Spikes of LI in 2008 and the spile of 2011 are obvious in Fig. 2a. Aperiodic oscillations of the indices of teleconnections are seen in Fig. 2b–f.

### Results of wavelet analysis

**Fig. 3:**
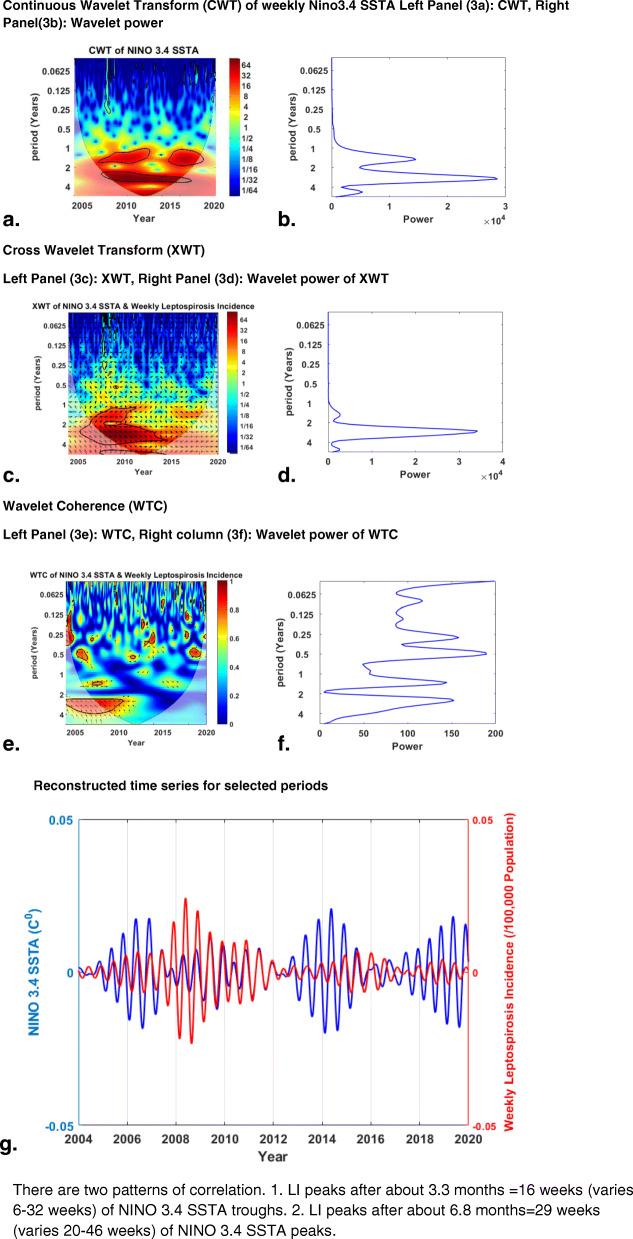
Results of wavelet analyses of weekly Niño 3.4 SSTA vs. weekly leptospirosis incidence for 2004–2019: (Panel **a**) continuous wavelet transform (CWT) variations; (Panel **b**) wavelet power of CWT; (Panel **c**) cross-wavelet transform (XWT) variations; (Panel **d**) wavelet power of XWT; (Panel **e**) wavelet coherence (WTC); (Panel **f**) wavelet power of WTC; and (Panel **g**) reconstructed time series for 2004–2019. There are two patterns of correlation. 1. LI peaks after about 3.3 months = 16 weeks (varies 6–32 weeks) of Niño 3.4 SSTA troughs. 2. LI peaks after about 6.8 months = 29 weeks (varies 20–46 weeks) of Niño 3.4 SSTA peaks

Figure 3 depicts the results of wavelet analysis of weekly Niño 3.4 anomaly vs. weekly LI results as a sample of our wavelet analysis results.

The explanation of Fig. 3 is very similar to the explanation of wavelet analysis results of two similar papers by us [3, 19]. Panel 3a of Fig. 3 shows the continuous wavelet transform of weekly Niño 3.4 SSTA, which expands the time series into time-frequency space, while panel 3b summarizes the power for each period. Panel 3c depicts the cross-wavelet transform of weekly Niño 3.4SSTA with weekly LI, whereas panel 4d illustrates the power for each period. As panels 3e and 3f depict, wavelet coherence is greatest between weekly LI and weekly Niño 3.4 anomaly for half-yearly (period) cycles. Color-coded panels on the right side of panels 3a, 3c, and 3e show the magnitudes of CWT, XWT, and WTC, in which dark blue and dark red indicate the lowest and highest, respectively. The thin U-shaped black lines in 3a, 3c, and 3e are the cone of influence. The thick black lines in panels 3a, 3c, and 3e are the 5% significance level using the red noise signal model. The arrows in panels 3c and 3e are vectors demonstrating the phase difference. A horizontal arrow pointing from left to right signifies the phase and an arrow pointing vertically upward means the second series lags behind the first by 90°.

Panel 3g is the reconstructed time series for 2004–2019.

Two correlation patterns between Niño 3.4 SSTA and LI are seen here. The mean time period between troughs of weekly Niño 3.4 SSTA and subsequent peaks in the weekly LI in this time series was 14 weeks (3.3 months) and that was the average lag period. In other correlation patterns, the mean time period between peaks of weekly Niño 3.4 SSTA and subsequent peaks in the weekly leptospirosis incidence in this time series was 23 weeks (5.4 months) and that was the average lag period. A document containing wavelet coherence results of all teleconnection indices with LI was added as a supplementary material.

### Results of time-lagged DCCA

Table 1 depicts the time-lagged detrended cross-correlation between teleconnection indices and LI with lag periods.

**Table 1:** Time-lagged detrended cross-correlation between teleconnection indices and LI with lag periods

Results of time-lagged DCCA analysis						
Lag(months)	Niño 3.4	SOI	MEI	Niño 4	EMI	DMI
**− 15**	− 0.002	0.034	0.099	− 0.059	− 0.028	0.105
**− 14**	− 0.032	0.097	0.088	− 0.135	− 0.117	− 0.031
**− 13**	0.039	0.016	0.022	− 0.014	− 0.055	− 0.176
**− 12**	0.105	− 0.094	− 0.018	0.150	0.094	− 0.124
**− 11**	0.050	− 0.089	− 0.039	0.140	0.121	0.098
**− 10**	− 0.047	0.002	− 0.017	− 0.007	0.059	0.297
**− 9**	− 0.079	0.032	0.040	− 0.091	− 0.078	0.147
**− 8**	− 0.071	− 0.038	0.036	− 0.114	− 0.155	− 0.100
**− 7**	− 0.004	− 0.015	− 0.027	− 0.036	− 0.081	− 0.223
**− 6**	0.084	0.005	− 0.069	0.106	0.036	− 0.162
**− 5**	0.101	0.040	− 0.055	0.176	0.125	0.036
**− 4**	0.012	0.114	− 0.032	0.065	0.031	0.145
**− 3**	− 0.114	0.042	0.014	− 0.137	− 0.127	0.095
**− 2**	− 0.140	0.035	0.056	− 0.233	− 0.164	− 0.050
**− 1**	− 0.006	− 0.031	0.067	− 0.088	− 0.015	− 0.141
**1**	0.134	− 0.026	***− 0.071***	0.144	0.062	0.033
**2**	0.053	***0.086***	***− 0.125***	− 0.006	***− 0.107***	***0.239***
**3**	− 0.060	***0.132***	***− 0.051***	***− 0.155***	***− 0.197***	***0.240***
**4**	***− 0.161***	0.017	0.017	***− 0.187***	***− 0.156***	0.015
**5**	***− 0.096***	− 0.088	0.007	− 0.095	− 0.040	− 0.227
**6**	0.008	− 0.045	− 0.029	0.025	0.044	− 0.196
**7**	0.046	0.021	***− 0.061***	0.058	0.085	0.040
**8**	− 0.038	0.065	***− 0.065***	− 0.056	0.007	0.199
**9**	***− 0.177***	0.082	− 0.038	***− 0.176***	− 0.077	0.051
**10**	***− 0.215***	0.069	− 0.044	***− 0.186***	− 0.083	− 0.158
**11**	− 0.084	0.041	− 0.033	− 0.080	− 0.022	− 0.224
**12**	0.048	− 0.036	− 0.001	0.069	0.117	− 0.075
**13**	0.091	− 0.059	− 0.042	0.125	0.182	0.133
**14**	0.009	− 0.042	− 0.009	0.080	0.123	0.236
**15**	− 0.045	− 0.031	0.083	0.036	0.011	0.122

Statistically significant correlations are italicized in Table 1. Except one, all those have occurred after a 2–4-month lag. Table 2 shows the summary of wavelet analysis and time-lagged detrended cross-correlation analysis results.

**Table 2:** Summary of wavelet analysis and time-lagged detrended cross-correlation analysis results

Teleconnection index vs leptospirosis incidence	Wavelet analysis results	Time-lagged detrended cross-correlation analysis results
**ENSO**		
Niño 4 SSTA vs leptospirosis incidence (LI)	Two patterns of correlation/1. LI peaks after about 14 weeks = 3.3months (varies 7–19 weeks) of Niño 4 SSTA troughs. 2. LI peaks after about 23 week = 5.4 months (varies 4–35 weeks) of Niño 4 SSTA peaks	LI rises significantly 3–4 months after decline of Niño 4 SSTA and again after 9–10 months (3-month lag CCF: 0.155, 4-month lag CCF: 0.187). Second pattern correlation after 6–7-month lag is not significant.
Niño 3.4 SSTA vs leptospirosis incidence (LI)	Two patterns of correlation/1. LI peaks after about 16 weeks = 3.7 months (varies 6-32 weeks) of Niño 3.4 SSTA troughs. 2. LI peaks after about 29 weeks = 6.8 months (varies 20–46 weeks) of Niño 3.4 SSTA peaks	LI rises significantly 4–5 months after decline of Niño 3.4 SSTA and again after 9–10 months (4-month lag CCF: 0.161, 5-month lag CCF: 0.096). Second pattern correlation after 6–7-month lag is not significant.
Multivariate ENSO Index (MEI) vs leptospirosis incidence (LI)	LI peaks after about 1.9 months (varies 1–3 months) of MEI Index troughs	LI rises significantly 1–3 months after decline of MEI and again after 7–8 months (2-month lag CCF: 0.125). Second pattern correlation after 4–5-month lag is not significant.
Southern Oscillation Index (SOI) vs leptospirosis incidence (LI)	LI peaks after about 2.2 months (varies 1-4 months) of SOI peaks	LI rises significantly 2–3 months after rise of SOI (2 months lag CCF: 0.086, 3 months lag CCF: 0.132)
**ENSO Modoki**		
ENSO Modoki Index vs leptospirosis incidence (LI)	LI peaks after about 11.5 months (varies 4.9–16.1 months) of ENSO Modoki peaks	LI rises significantly 2–4 months after decline of MEI (3-month lag CCF: 0.197)
**IOD**		
Indian Ocean Dipole Mode Index (DMI) vs leptospirosis incidence (LI)	LI peaks after about 24 weeks = 5.6 months (range varies 6–40 weeks) of DMI peaks	LI rises significantly 2–3 months after rise of DMI (2-month lag CCF: 0.239, 3-month lag CCF: 0.240)

## Discussion

Broadly, there is an agreement between the results of wavelet analysis and time-lagged DCCA. The wavelet analysis method was successfully employed to identify correlation patterns between teleconnections, weather, and health outcomes [3, 5, 17–19]. That method’s inability to determine magnitudes of correlations can be compensated and that results can be crosschecked by using parallel time-lagged DCCA analysis methods in similar future studies as we did.

### How correlations between teleconnections and rainfall and other meteorological parameters modulate LI in Kandy

The detection of two correlation patterns between three indices of ENSO and LI by wavelet analysis is a good evidence of the existence of two correlation patterns. However, the second correlation pattern detected by time-lagged DCCA was not significant. The first correlation pattern (LI peaks after SSTA troughs with a lag) detected by wavelet analysis agrees with the time-lagged DCCA findings. Two correlation patterns between ENSO and rainfall of Sri Lanka were demonstrated by past studies [10, 15, 16, 26]. La Ni*ñ*a (El Niño) events enhance (reduce) the rainfall in Sri Lanka during the First Inter-Monsoon (April) and South West Monsoon (May–September) and North East Monsoon (December–February) [10, 26, 27]. During El Ni*ñ*o events, rainfall increases for the first 3 months of the main agriculture season named the Maha season (October–December) and decreases during the last 3 months (January–March) [10, 26, 27]. A recent study showed a reduction of rainfall during La Ni*ñ*as during the Second Inter-Monsoon period (mainly October) [10]. Rainfall data of some weather stations of the Kandy district also were used in those studies. Rainfall in Kandy was correlated with LI [5]. Those past findings increase the trustworthiness of our results. During La Ni*ñ*a (cold phases of ENSO) Niño 3.4 and 4 indices become strongly negative and the SOI becomes strongly positive. Our Table 2 that summarizes the correlation pattern of all those ENSO indices portrays upsurges of LI following La Ni*ñ*as. Both analysis methods employed reveal this. There is a paucity of past studies on modulation of temperature, humidity, and other weather parameters of Sri Lanka by ENSO. Nonetheless, it is well known that all meteorological parameters are interrelated and changes in ocean temperatures usually change atmospheric temperatures in island nations. We have demonstrated peaks of rainfall (in mm), the count of wet days per week and the number of days with > 100 mm of rainfall per week were correlated with peaks of LI in the Kandy district during 2006–2015 [5]. Other meteorological parameters were also correlated with the LI of Kandy [5]. All above considered, we can conclude that ENSO modulates LI of the Kandy district via modulating the rainfall and possibly other meteorological parameters especially during the cold phase of ENSO.

Strong El Ni*ñ*o Modoki events caused lower rainfall during the months of February and July and above normal rainfall during the months of August, September, and October in Sri Lanka in a 61-year-long study [16]. Strong La Ni*ñ*a Modoki conditions resulted in higher rainfall in January and February and below-normal rainfall in May and October [16]. Thus, it is possible for two correlation patterns to exist between EMI and LI in Kandy. Our time-lagged DCCA results show a rise of LI following La Ni*ñ*a Modoki events. Wavelet results show the opposite with too long lag periods compared to other lag periods in Table 2. We do not know the exact reason but we think this discord in results is due to differences in two analysis methods. It is possible that two patterns of correlation exist and each analysis method detected only one correlation pattern. Such different results in two analysis methods were there in some similar past studies [3, 19]. ENSO Modoki modulates the rainfall in Sri Lanka and the rainfall was correlated with LI in Kandy [5, 16]. Hence, we believe that the LI of Kandy is more likely to be modulated by ENSO Modoki than not. However, further studies are necessary to confirm this and to determine the effects of ENSO Modoki on the LI of Sri Lanka.

LI is also influenced by several other meteorological parameters than rain and many non-weather factors as well [1–5, 8]. The strength of the influence of those factors on LI varies from area to area. Thus, we believe we cannot generalize correlation patterns between teleconnections and LI we derived to the whole Sri Lanka without further studies.

### Evidence for two patterns of correlation between teleconnection indices and LI from past studies

Two correlation patterns between another ENSO index (Oceanic Niño Index) and LI were detected in Columbia in the past [28]. Seventeen municipalities of Columbia had a rise in leptospirosis cases during La Ni*ñ*a periods. Of those, seven additionally had an increase in leptospirosis during the El Ni*ñ*o month [28]. In comparison, there were two patterns of correlation between DMI and LI in the Hambantota district of Sri Lanka but there was no clear correlation between Niño SSTA and LI in Hambantota [3]. Interestingly, here in the Kandy district, there was only one correlation pattern between DMI and LI. Positive DMI was shown to enhance rainfall in Sri Lanka during Maha growing season and that explains the subsequent rise of LI [15, 29].

### Which teleconnection index is best correlated with the LI in Kandy

Out of indices of three teleconnections studied, the index of IOD (DMI) had the highest magnitude correlation with the LI of Kandy. Therefore, IOD may be the teleconnection that matters most to the LI of Kandy.

There are different views in the climate science community about which ENSO index best describes ENSO phases and strength [9]. ENSO signature changes with the season and the location [9]. Some experts suggest a pressure anomaly index like SOI might be more suitable for Southern Asia (Sri Lanka) to study ENSO-related health outcomes [9]. They also state, to determine rainfall- and temperature-related health outcomes like LI, a SSTA index may be appropriate for places close to Niño oceanic regions [9]. We used SOI as well as Niño 3.4 and Niño 4 indices estimated considering Niño oceanic regions closer to Sri Lanka. We detected LI correlated with all indices of ENSO we studied.

Correlation patterns of SSTA indices of ENSO (Niño 3.4, Niño4), atmospheric pressure anomaly index of the ENSO (SOI), and Multivariate ENSO Index (MEI) with the LI of Kandy, demonstrated that ENSO modulates LI of the Kandy district in the same way. Similar results by a study of different indices confirm ENSO’s pattern of influence on the LI of Kandy. During the negative phase of Niño 3.4, Niño 4, and MEI indices of ENSO (La Ni*ñ*a), SOI is in the positive phase and vice versa happens [9]. Therefore, SOI among the ENSO indices studied is expected to show a statistically positive correlation, when other (ENSO) indices have statistically negative association. Our results (Table 2) agree with that correlation pattern and it further supports that the LI of Kandy is correlated with ENSO. It is also intriguing to note similar correlation patterns shown by SOI and DMI (SSTA index of IOD) with the LI especially in DCCA results, but we are unable to give an exact reason for that.

Out of the ENSO indices, Niño 4 shows the highest magnitude correlation with LI after a 3–4-month lag (Table 2) and therefore may be the most suitable ENSO index to predict the rise of LI in Kandy. Niño 4 is computed considering an area closer to Sri Lanka compared to areas considered for estimation of Niño 3.4 and SOI. We speculate that might be a reason for the stronger correlation. MEI is a more holistic depiction of ENSO created by combining five variables of both SSTA and atmospheric components of the ENSO [12, 27]. Contrary to our expectations, its correlation with the LI of Kandy was weaker than the correlations of orthodox Niño indices and SOI. One study that included data of the Peradeniya weather station of the Kandy district has demonstrated that MEI’s correlation with rainfall anomalies in Sri Lanka usually is stronger compared to Niño SSTA indices during 1950–2013 [27]. Nevertheless, during the North East monsoon season, rainfall anomalies had slightly stronger correlation with Niño SSTA [27]. LI in Kandy is usually very high after the onset of the North East monsoon season [5]. The same study showed DMI’s correlation with rainfall anomalies is weaker than that of Niño indices and MEI except during the South West monsoon season [27]. Those findings indicate stronger influence on changes of the LI of Kandy by factors other than rainfall modulation by teleconnections.

### Comparison of our results with that of similar past studies and discussion

The first demonstration that ENSO indices have a strong correlation with LI by a time series analysis was in 2014 [30]. There were previous reports on ENSO influencing LI, but those authors have not studied the correlation between time series of indices of ENSO and LI [30]. Authors of reference 30 have found that La Ni*ñ*a periods were linked to high rainfall, and both of these factors were, in turn, associated with outbreaks of leptospirosis in New Caledonia like in our study [30]. A few similar long-term studies followed that [3, 28]. More similar studies will improve our understanding of the ENSO and other teleconnections’ correlation with LI. That is important as leptospirosis is already a leading zoonotic cause of morbidity and mortality and more frequent extreme El Ni*ñ*o and IOD events are likely to happen with the ongoing climate changes [6, 31, 32]. ENSO indices were correlated with the incidences of diverse infectious disease in the past illustrating how ENSO influence transmission mechanisms of various infectious tropical diseases by modulating the local weather. That includes arthropod-borne parasitic infections malaria and leishmaniasis [33, 34], mosquito-borne viral infections like dengue [17], robovirus diseases like hantavirus infections [35]; hand, foot, and mouth disease, a viral disease transmitted by feco-oral route as well as respiratory route [36]; and feco-orally transmitted bacterial infection cholera [18]. IOD was described to modulate the incidence of dengue [17]; chikungunya, an arboviral disease [37]; and cholera [18].

However, generally, there is a paucity of studies on teleconnections’ influence on weather-sensitive diseases. There is one past study that looked for the influence of all three teleconnections considered in our study on malaria in Papua New Guinea [33]. They found generally a negative correlation between malaria cases and Niño 3.4, EMI, and DMI [33]. Considering the heterogeneous correlation patterns in various areas of that country and different levels of influence on malaria incidence by confounders, those authors recommend location-specific studies to understand teleconnections’ influence on malaria. A study from Columbia showed heterogeneous correlation patterns in different localities between monthly leptospirosis cases and an index of ENSO [28]. Considering the results of the present study and our past study in the Hambantota district, we also think location-specific studies are needed to get a good idea of teleconnections’ LI correlation of an area [3].

A negative correlation was detected between the monthly MEI index of ENSO and LI with a a 7-month lag in Thailand 2000–2014 [38]. Those researchers attribute flooding associated with La Ni*ñ*as to the rise of LI [38]. Temperature, relative humidity, and SOI, but not rainfall, were significantly and interestingly independently associated with dengue cases in Singapore [39]. The 2004 leptospirosis outbreak in Guadeloupe in an unusual season was attributed to the combination of locally uncommon leptospira serogroup becoming common among rodents who released them to the environment and weather conducive for leptospirosis transmission as a result of El Ni*ñ*o [40]. Hemorrhagic fever with renal syndrome (HFRS), a disease that has a clinical picture similar to severe leptospirosis, is caused by the hanta virus [41]. People contract HFRS also mainly from the urine of infected rodents [41]. However, in contrast to leptospires, hanta viruses infect people mainly when they inhale aerosolized viruses [41]. A study from China shows the density of reservoir rodents hosting the hanta virus and MEI (ENSO) had the greatest effect on the transmission of HFRS out of several factors studied [35]. Those researchers believe climatic and environmental factors do not play a direct role in transmission but they modulate HFRS incidence via their influence on rodent density [35]. We could not find any information about serial changes of rodent density of Kandy 2005–2019 to study ENSO rodent density correlation and compare. A research team of South Korea has studied how ENSO’s effects on an infection transmission in faraway places affect their country. They have studied correlation between ENSO and the count of imported Shigellosis cases to their country [42]. 87.1% of imported cases of shigellosis were from South and Southeast Asia [42]. Shigellosis is a water-borne bacterial diarrheal disease. They state that heavy rains in South and Southeast Asia following La Ni*ñ*a conditions increase visitors with Shigellosis to South Korea [42]. Another study from Colombia demonstrated rise of leptospirosis following increase of rainfall as a result of some (not all) La Ni*ñ*as [43]. We also detected large peaks of LI following some but not all troughs of Niño indices. Analysis with long-term data series that include more than one ENSO event gives better evidence of ENSO disease correlations [44]. During the period of our study, there were a few ENSO cycles and that enhances the fidelity of our results. Regarding ENSO, we conclude that in conducive weather conditions for leptospirosis transmission created by La Ni*ñ*as, LI rises after a lag and if other factors also contribute to transmission, major leptospirosis outbreaks befall in Kandy like in 2008 [3]. The peak of LI occurs after El Niños with longer lags.

### Discussion of 2008 and 2011 outbreaks of leptospirosis

The two major leptospirosis outbreaks of Sri Lanka in this century happened in 2008 and 2011 [3, 8]. Those can be seen in Fig. 2a. Corresponding 2007–2008 and 2010–2012 La Ni*ñ*as can be seen in Fig. 2b–e. The Kandy district was severely affected in the 2008 outbreak. In 2008, Kandy district LI was very high from April to the end of June; the usual peak during the last quarter of the year was also augmented. LI is modulated by many important non-weather factors as well [1, 3, 5]. If those factors also simultaneously significantly contribute to the spread of leptospirosis, a major outbreak results in conducive weather conditions created with contributions from teleconnections, akin to the classic Swiss cheese model [3].

### Relevance of our findings to Leptospirosis preventive work

Notified LI (which are moderate or severe cases) in the Kandy district is high, and considering unreported cases, the true incidence may be much higher [5, 6, 8]. Most leptospirosis deaths in Sri Lanka are men of working age and are breadwinners of their families [8]. Hence, both primary and secondary prevention are important. Information on extreme teleconnection events is freely available online from the sources mentioned in our data statement. Preventive health authorities of Sri Lanka can regularly monitor those sources, detect extremes (like La Ni*ñ*as, highly positive DMI, and La Ni*ñ*a Modokis), and escalate preventive work during the lag periods. We recommend that as it will enable us to preempt impending LI peaks. Enhancement of preventive methods like education of the general public and targeting vulnerable populations regarding avoidance of exposure, personal protection, and basic sanitation, taking chemoprophylaxis during high-risk activities like working in rice paddies, immunization of livestock and dogs, rodent control, and the importance of going to a hospital early if they have symptoms would be useful [1–5]. However, the problem we see is most healthcare workers we know including some holding top posts (local and abroad) are unaware of the significance of teleconnections for LI (some of them are not aware of the existence of teleconnections). We hope publications like this would contribute to increase awareness of healthcare workers especially doctors who hold key posts. We think the process we propose to blunt LI peaks that ensue after extreme teleconnection events can be applied elsewhere after determining the correlation pattern between teleconnection indices and local LI.

### Limitations

Unmeasured important confounders could have affected our results; such confounders include leptospirosis outbreaks among animals especially if that release leptospires of uncommon serotypes to the environment, fluctuations in local rodent density, and socio-demographic factors of the population [1–4, 40]. After practicing medicine for more than two decades in Kandy, the first and fifth authors’ understanding is notified leptospirosis cases are almost always hospitalized cases. Mild infections and asymptomatic cases do not get hospitalized, and despite legal provisions, even some hospitalized cases are not notified. Periodic reminders to clinicians about the necessity of notification may help to increase notifications. According to one study, the degree of modulation of rainfall by ENSO in three locations (Peradeniya, Hanthana, and Kundasale) in the Kandy district is not the same [10]. That indicates the modulation of LI in the Kandy district by teleconnections is unlikely to be uniform. The first author had seen a few patients who were likely to have contracted leptospirosis indoors, for example, by working at rat-infested wholesale stores in the Kandy city. The influence of local weather is lesser in such transmissions. Nonetheless, such cases are a minority.

## Conclusions

The results of wavelet analysis and time-lagged DCCA methods generally agree. A past study has demonstrated rainfall and other meteorological parameters modulate LI in Kandy. Those indicate that ENSO and IOD modulate LI in Kandy by modulating local rainfall and probably other weather parameters. Considering the different results of the two analysis methods, we recommend further studies about ENSO Modoki and LI correlation in Sri Lanka. Monitoring for extreme teleconnection events and enhancing preventive measures during lag periods in Sri Lanka can blunt LI peaks that may follow.

## Supplementary Information

**Additional file 1.**

## Data Availability

We have used secondary data. We do not own them and they are available from the following owners of data. The counts of reported cases of leptospirosis from Kandy were obtained from weekly epidemiology reports of the Ministry of Health of Sri Lanka: Epidemiology Unit, Ministry of Health of Sri Lanka, 231 De Saram Place, Colombo 10, Sri Lanka. Midyear population data was obtained from the Sri Lanka Department of Census & Statistics, “Sankayana Mandiraya” No. 306/71, Polduwa Road, Battaramulla, Sri Lanka. Part of that data is available online as well: http://www.statistics.gov.lk/PopHouSat/VitalStatistics/MidYearPopulation/Mid-year%20population%20by%20district.pdf . Monthly Niño 3.4 and Niño 4 SST anomaly indices, plus monthly DMI data is available online from the NOAA Physical Sciences Laboratory, National Oceanic and Atmospheric Administration of the United States: https://www.psl.noaa.gov/gcos_wgsp/Timeseries/, https://psl.noaa.gov/enso/mei/, https://www.ncdc.noaa.gov/teleconnections/enso/indicators/soi/. Weekly DMI anomaly data and monthly EMI data were kindly provided by Dr.Takeshi Doi of the Japan Agency for Marine-Earth Science and Technology (JAMSTEC).

## Abbreviations

LI: Leptospirosis incidence
ENSO: El Ni*ñ*o Southern Oscillation
IOD: Indian Ocean Dipole
SOI: Southern Oscillation Index
MEI: Multivariate ENSO Index
DMI: Dipole Mode Index
EMI: El Ni*ñ*o Modiki Index
DCCA: Detrended cross-correlation analysis
SST: Sea surface temperature
SSTA: Sea surface temperature anomalies
NOAA: National Oceanic and Atmospheric Administration
JAMSTEC: Japan Agency for Marine-Earth Science and Technology
XWT: Cross-wavelet transform
WTC: Wavelet coherence
CWT: Continuous wavelet transform
HFRS: Hemorrhagic fever with renal syndrome
