# Supplementary material for: The correlation between three teleconnections and leptospirosis incidence in the Kandy District, Sri Lanka, 2004–2019
Source: Trop Med Health. 2021 May 26;49:43. doi: 10.1186/s41182-021-00325-z (PMC8152333; doi:10.1186/s41182-021-00325-z)
Supplement: Supplementary file 1 — Additional file 1. [file 41182_2021_325_MOESM1_ESM.docx]

**Wavelet Coherence (WTC)-** **NINO 4 SSTA vs Leptospirosis incidence (LI)**

| 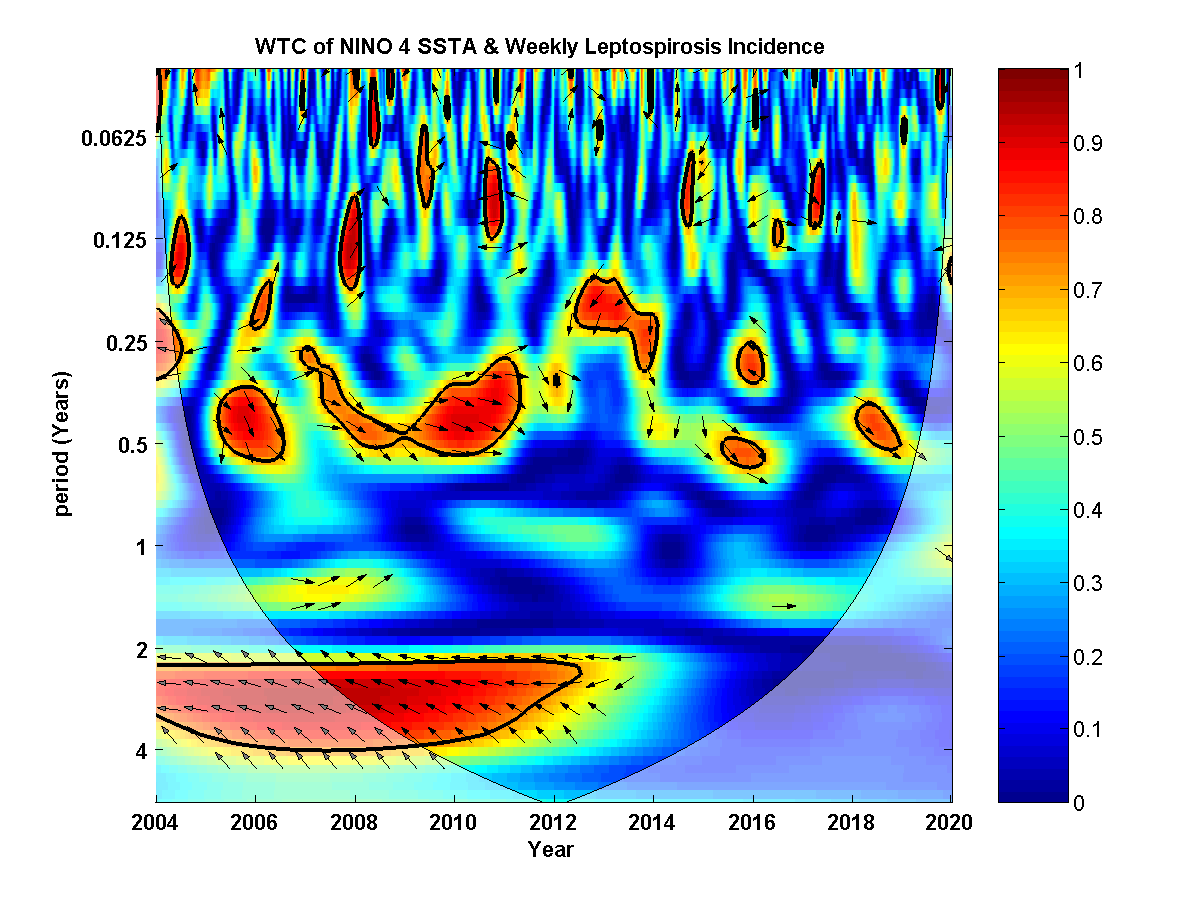 | 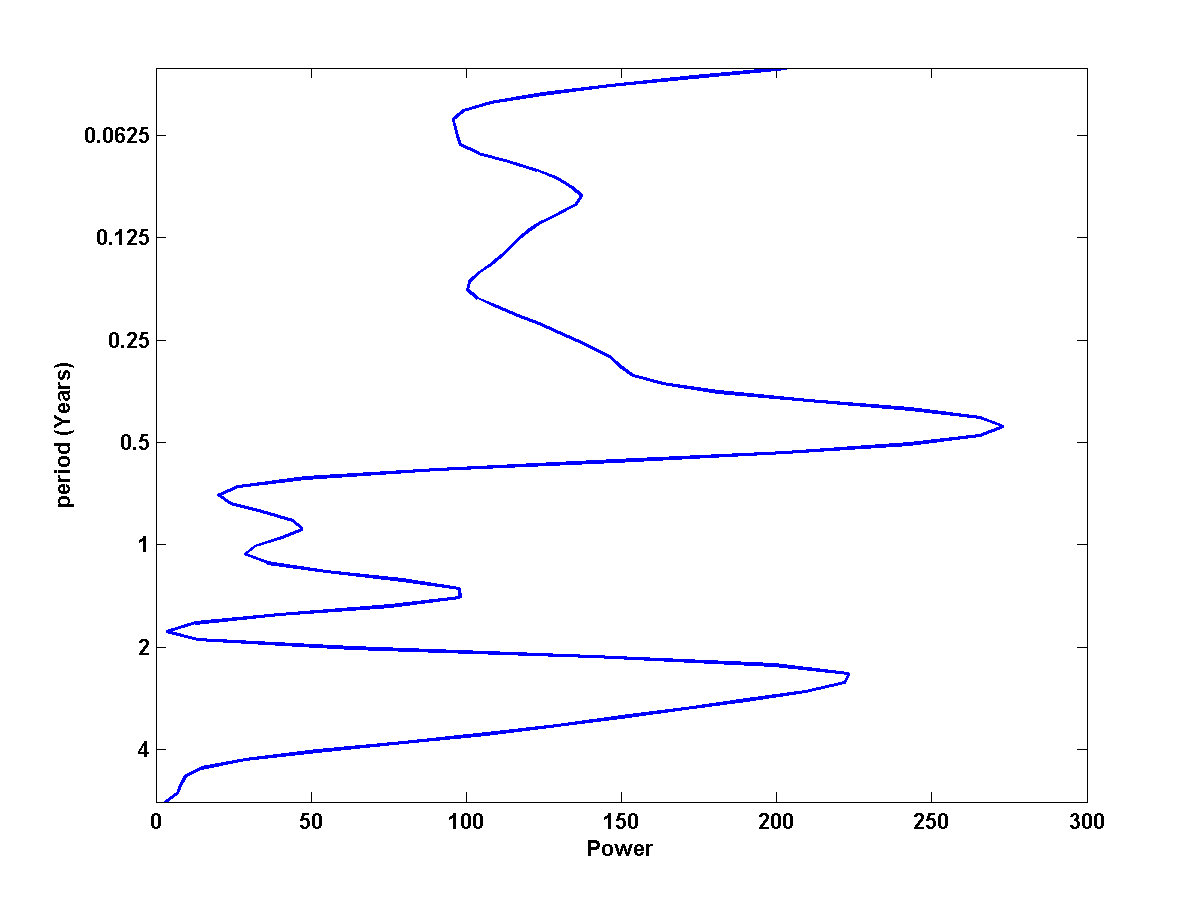 |
| --- | --- |

**Wavelet Coherence variations**

Left column: WTC, Right column: Wavelet power

**Wavelet Coherence (WTC)-** **NINO 3.4 SSTA vs Leptospirosis incidence (LI)**

| 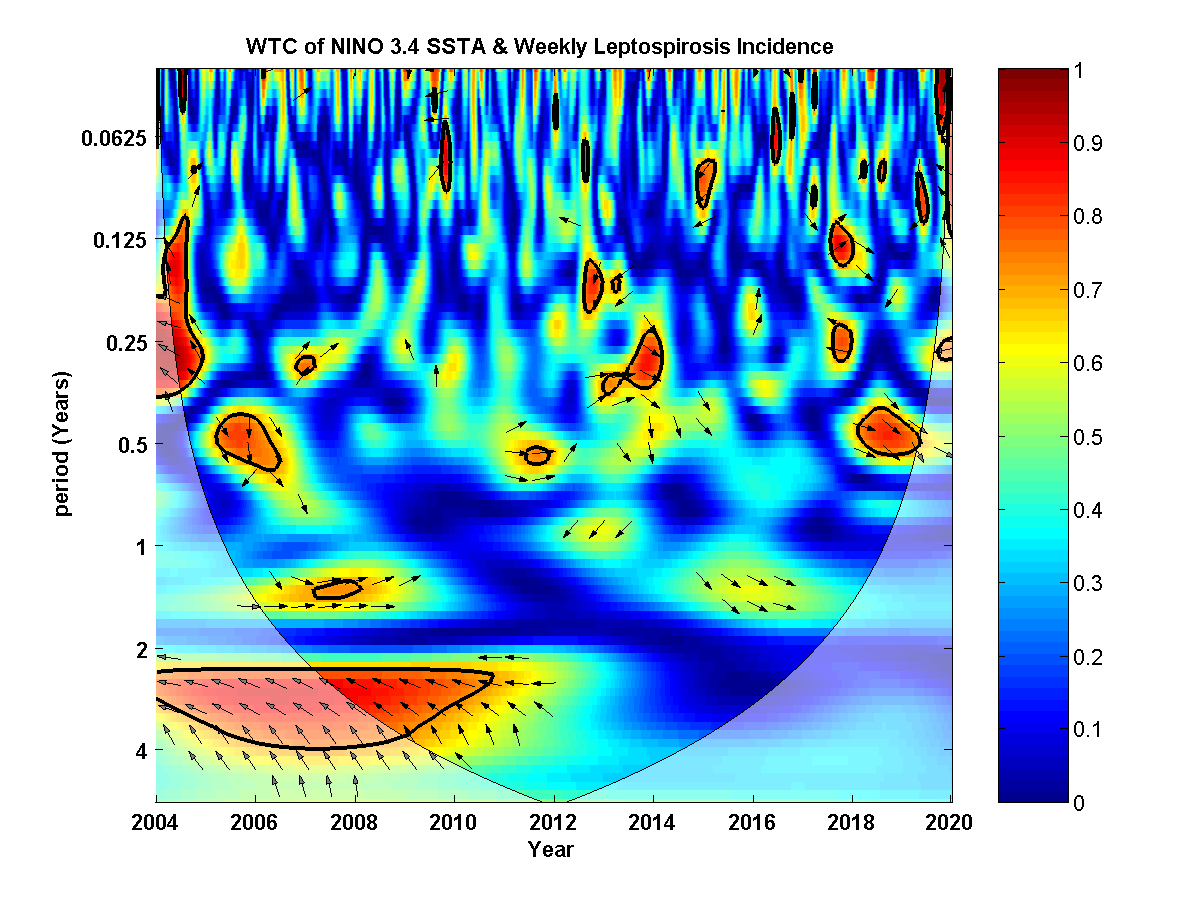 | 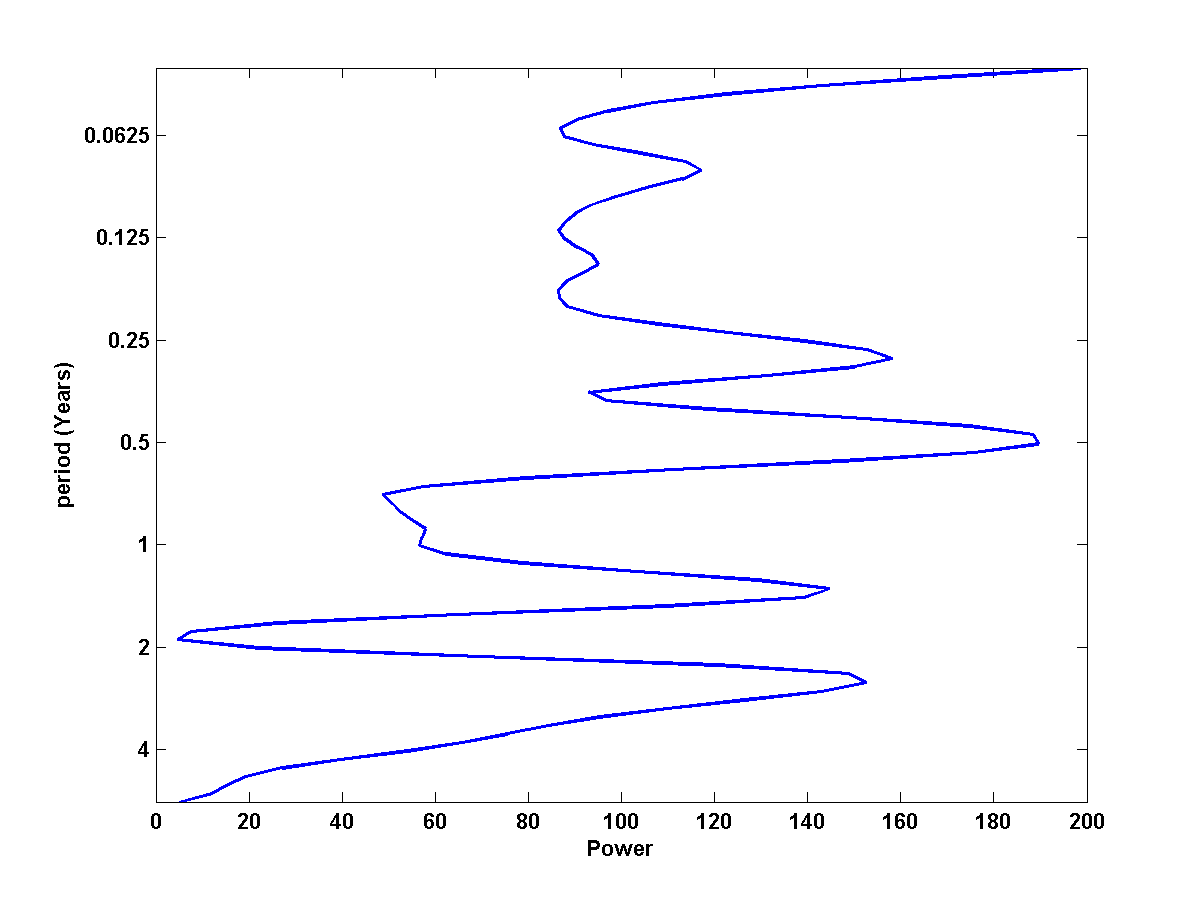 |
| --- | --- |

**Wavelet Coherence variations**

Left column: WTC, Right column: Wavelet power

**Wavelet Coherence (WTC)- SOI vs Leptospirosis incidence (LI)**


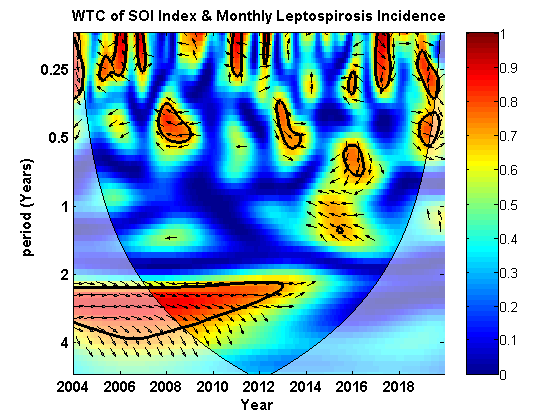

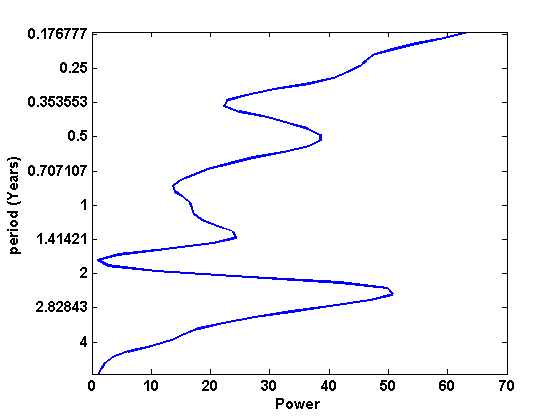


**Wavelet Coherence (WTC)- MEI vs Leptospirosis incidence (LI)**

**
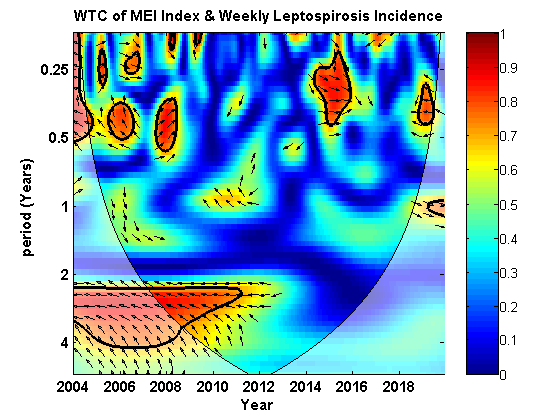

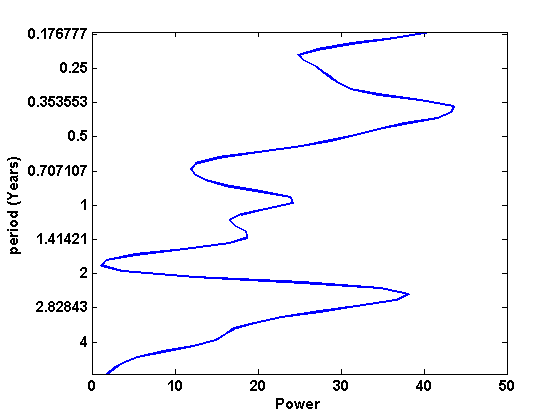
**

**Wavelet Coherence (WTC)-** **Indian Ocean Dipole Mode index (DMI) vs Leptospirosis incidence (LI)**

| 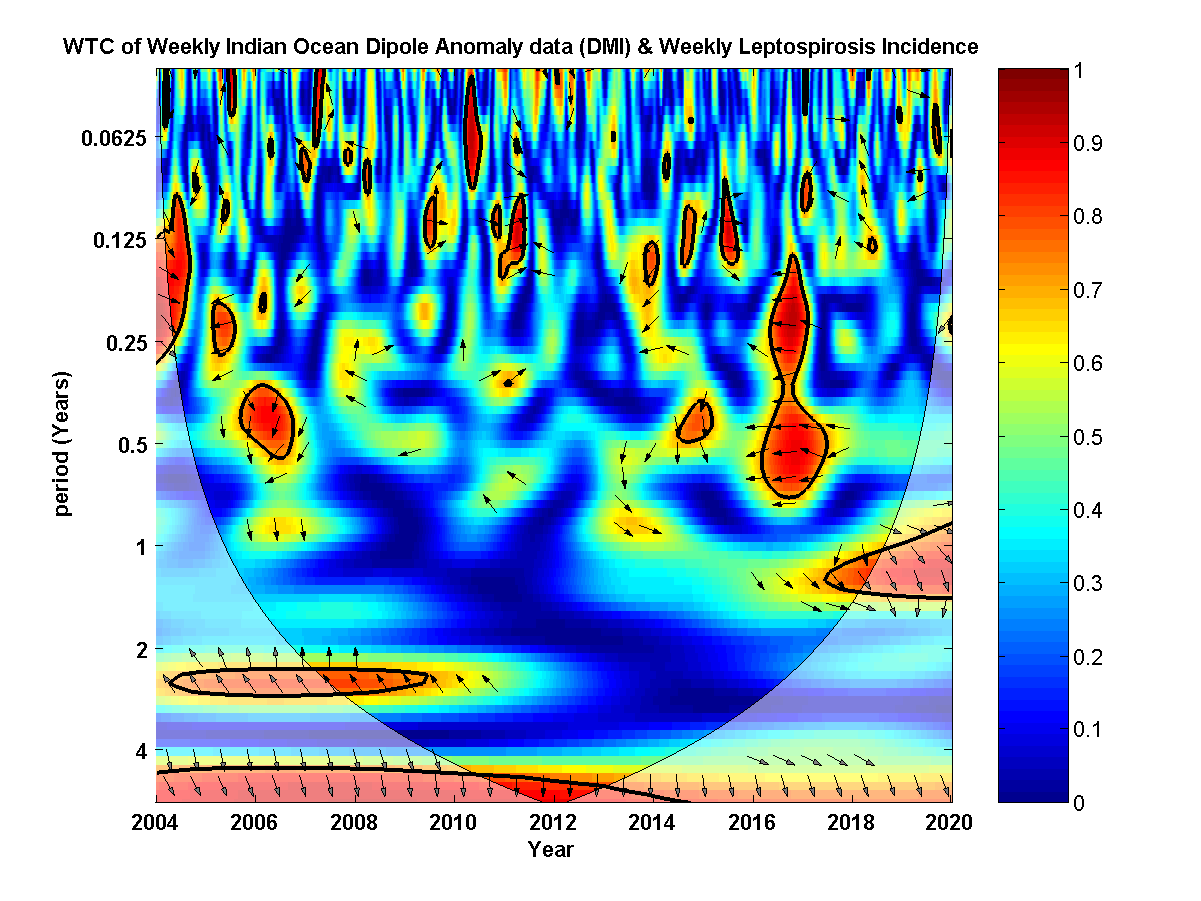 | 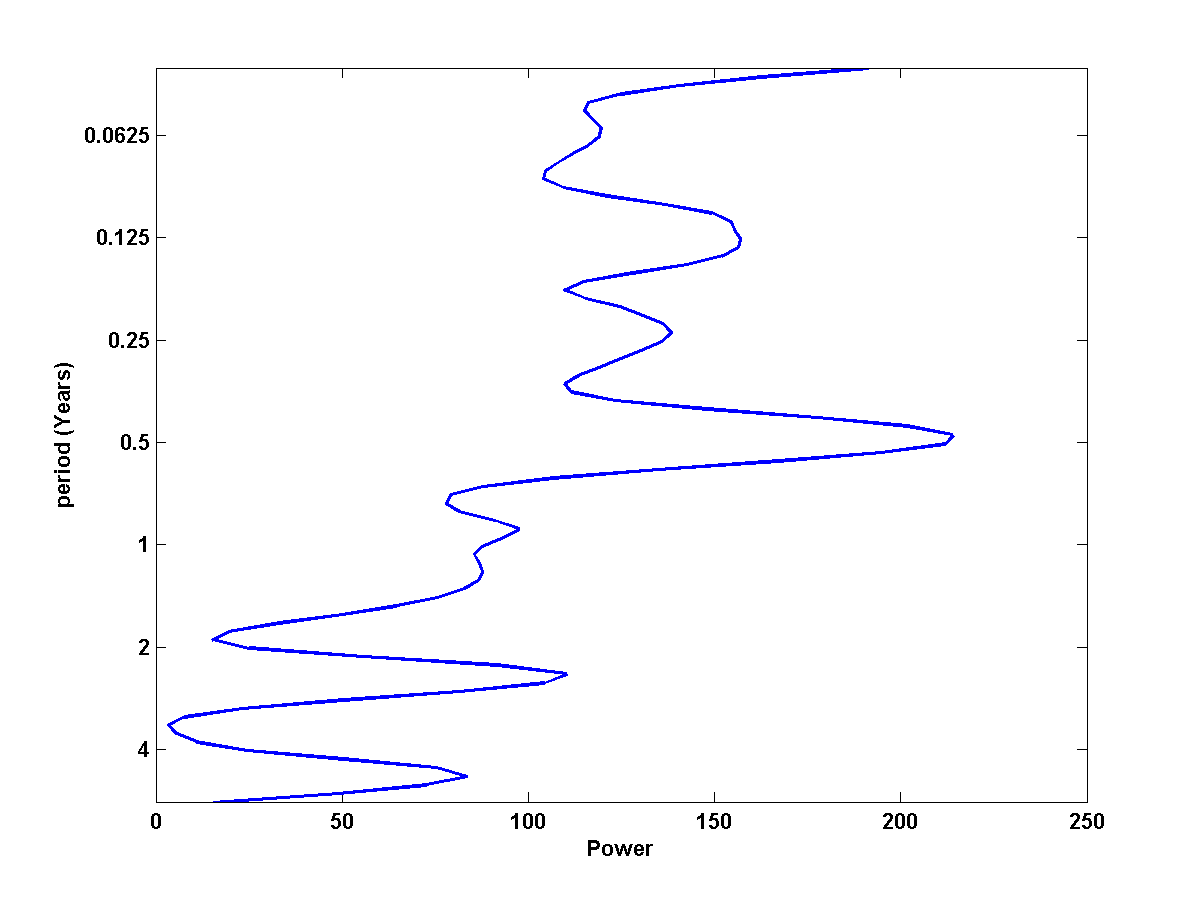 |
| --- | --- |

**Wavelet Coherence variations**

Left column: WTC, Right column: Wavelet power

**Wavelet Coherence (WTC)- El Nino Modoki Index (EMI) vs Leptospirosis incidence (LI)**

**
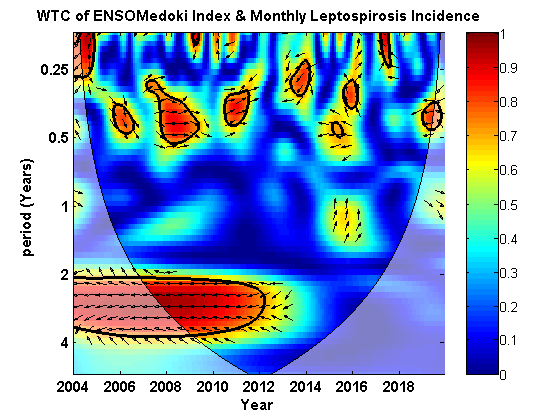

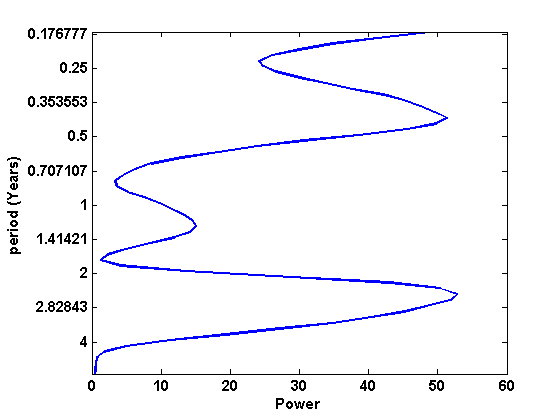
**
